# Reversible Stability of Emulsion and Polymer Latex Controlled by Oligochitosan and CO_2_

**DOI:** 10.3390/polym10121352

**Published:** 2018-12-06

**Authors:** Liang Li, Na Guo, Xiao Zhang, Wen Ou, Shengcai Yang, Xin Su, Yujun Feng

**Affiliations:** 1 Unconventional Petroleum Research Institute, China University of Petroleum, Beijing 102249, China; liliang.xbsj@sinopec.com; 2 Sinopec Northwest China Oilfield Company, Urumqi 830011, China; guon.xbsj@sinopec.com (N.G.); z505x@163.com (X.Z.); 3 Polymer Research Institute, State Key Laboratory of Polymer Materials Engineering, Sichuan University, Chengdu 610065, China; wen123ou@163.com (W.O.); ysc330@126.com (S.Y.)

**Keywords:** CO_2_, oligochitosan, salt addition, latex, emulsion

## Abstract

The addition of salt to a colloid solution ensures that emulsions can be easily separated into two phases and that polymer latexes can be coagulated. The switchable stability of emulsions and polymer latexes would improve the properties for their current applications. A switchable process of salt addition can be achieved using CO_2_ and switchable water, and it is a novel, benign approach to achieving a switchable ionic strength in an aqueous solution. However, the problem associated with switchable water is that its additives are all synthetic tertiary amines, most of which are harmful to human beings and the environment. Oligochitosan, as a natural product, can also be used as a switchable water additive. In this paper, a new switchable water system using oligochitosan to change the ionic strength was explored for use in several potential industrial applications. The conductivity of the aqueous solution of oligochitosan (0.2 wt.%) was switched from 0.2 to 331 μS/cm through the addition and removal of CO_2_. Oligochitosan and CO_2_ were successfully utilized to reversibly break a crude oil emulsion. Polystyrene (PS) latexes could also be reversibly destabilized; the zeta potential of the PS latex changed between −5.8 and −45.2 mV in the absence and presence of CO_2_ after oligochitosan was dissolved in the PS latex. The use of oligochitosan is a more environmentally friendly means for reversibly separating colloid solutions.

## 1. Introduction

Salt addition is one of the most common post-treatments for emulsions and polymer latexes, where it is used to recover and use hydrophobic material dispersed in the colloids [1]. The addition of common inorganic salt to a colloid solution ensures that emulsions can be easily separated into two phases and polymer latexes can be coagulated. One of the application examples for emulsions is the emulsification of crude oil, which is critical for its pipeline transportation. Crude oil emulsions with low viscosity reduce both cost of delivery and rate of corrosion in the pipeline transportation system. Crude oil and water can be separated at the end of transport through salt addition, and the crude oil that is delivered to the destination could be treated further, such as in a petroleum refinery. For polymer latexes synthesized from emulsion polymerizations, most applications involve obtaining polymer resins or powders from the aggregation of latexes after salt addition, but some applications require a latex form, such as in coatings and adhesives; it is hypothesized that if the redispersion of the polymer powders in water is obtained, it would be helpful to transport latexes in a polymer powder form and redisperse the dry powder in water for final use. The basic principle of salt addition is that the enhanced ionic strength collapses the electrical double layer of dispersed phases, such as those in emulsion droplets and latex particles, resulting in their aggregation and precipitation based on the DLVO theory which displays the explanation of the stability of colloidal suspension [2]. However, it is difficult to reuse the added inorganic salt [1]. If the added salt possesses reversible properties, the switchable stability of emulsions and polymer latexes could be accomplished.

CO_2_ responsive materials [3,4,5,6,7,8,9,10] have attracted increasing attention because of their astonishing abilities in various applications, such as commanding the surface activity of surfactants [3], switching the hydrophilicity of substrate surfaces [4], changing the polarity of solvents [6], controlling the stability of polymer latexes [5,7,8,9] and even dominating the size of hydrogels [10]. The mechanism of CO_2_ switching involves the reversible reaction of CO_2_ and switchable groups such as amines [4,5]. The utilization of CO_2_ as a trigger can generate many switching cycles without the accumulation of any byproduct. Furthermore, CO_2_ has the advantage of being a cheap, safe, and efficient trigger for switching the properties of CO_2_ responsive materials [5].

Recently, to improve the process of salt addition, Jessop and coworkers reported a new method called “switchable water,” which is an example of a CO_2_ responsive material [11]. Switchable water is an innovative mixture of water and water-soluble amine, where the removal or addition of CO_2_ switches the ionic strength of the aqueous solution to expected values [11]. The dissolved water-soluble amine has a very low ionic strength in the absence of CO_2_. However, after the addition of CO_2_, the amine is protonated and changed into a bicarbonate salt, which causes the ionic strength to rise sharply. The application of switchable water allows reversible salt addition approaches, which allow the water to be recycled, reducing energy consumption.

**Scheme 1:**
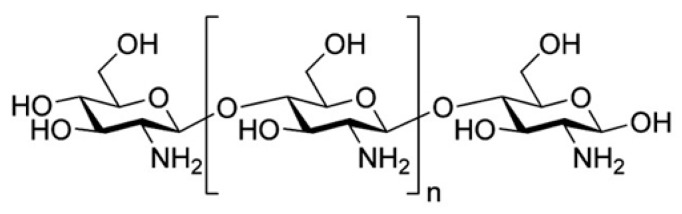
Molecular structure of oligochitosan. The repeating number n is located in a range from 0 to 18.

Although switchable water can be used in green chemistry, some studies have demonstrated that switchable water with tertiary amines is not an asset in relieving toxic effects in comparison to common compounds or methods [12]. One of the main targets of green chemistry is developing environmentally friendly compounds that can replace existing chemicals and alleviate their negative influence on nature and humans. Therefore, effort should be committed to lessening the existing toxicity of switchable water additives. Because all of the switchable water additives are amines, which are the CO_2_-switchable groups, we considered whether it would possible to select a natural amine as a candidate for a switchable water additive rather than an artificial or synthetic one. Although alkaloids [13] and nucleobases [14] would be great choices, only oligochitosan [15] was selected in our research. This was because oligochitosan is more common and cheaper, and its molecular structure with polyhydroxy groups shows that it should be more water soluble than the former two. Oligochitosan is an amino sugar and one of the most abundant monosaccharides (Scheme 1). It is produced commercially through the hydrolysis of chitosan and is one of the most common dietary supplements [16]. There is no doubt that oligochitosan is healthy and safe for both humans and the environment.

Herein, we propose a new switchable water additive, oligochitosan, whose aqueous solution has a low ionic strength in the absence of CO_2_. However, after the introduction of CO_2_, these charged amine species increase the ionic strength of the solution. The ionic strength can be controlled through the addition and removal of CO_2_. In addition to testing the conductivity to confirm the reversibility, oligochitosan was added to a crude oil emulsion and polystyrene (PS) latex. This proved that oligochitosan acted as a switchable water additive in colloid solutions, and those mixtures had reversible stability through multiple switching cycles.

## 2. Materials and Methods

### 2.1. Materials

Oligochitosan (molecular weight = 1227, PDI = 1.04) was purchased from the Chunxin BioTech Company (Shijiazhuang, China). Sodium dodecyl sulfate (SDS) and styrene (99%) were purchased from Sigma-Aldrich. The styrene was purified using inhibitor removal columns (Sigma-Aldrich, St. Louis, MO, USA) before polymerization. The initiator 2,2′-azobis(isobutyronitrile) (AIBN) was bought from TCI Co., Ltd. (Tokyo, Japan). CO_2_ and N_2_ with purities of 99.998% were obtained from the Tianyuan Gas Company (Chengdu, China). The deionized water used in this study (conductivity, κ = 18.25 μS·cm^−1^) was triple distilled using a quartz water purification system. Crude oil was supplied from the Sinopec Northwest China Oilfield Company (Urumqi, China).

### 2.2. Preparation of Polystyrene Latex

Styrene (15 mL), AIBN (0.1 g), and SDS (0.5 g) were mixed in deionized water (50 mL). The mixture was stirred continuously at 400 rpm under a N_2_ atmosphere for 30 min. The polymerization started and the reaction was controlled at 75 °C for 7 h.

### 2.3. Persistency Tests of Target Colloid Solutions Triggered by CO_2_

The target colloid solutions included a crude oil emulsion and PS latex. The crude oil emulsion was produced by mixing crude oil (20.0 g), water (50.0 mL), and SDS (1.0 g). The PS latex was used immediately after polymerization.

The target colloid solution was then destabilized by the continuous sparging of CO_2_ (90 mL·min^−1^) through the solution within 20 min at 25 °C. Then, the target colloid solution was allowed to separate into two phases. To homogenize the separate phases into a stable colloidal solution again, N_2_ (90 mL·min^−1^) was sparged into the solution for 20 min to release the CO_2_ through shaking by hand [16]. The flow rate of the CO_2_ and N_2_ was monitored by a flowmeter.

The desired amount of oligochitosan was added to one of the target solutions stored in a glass vial using a magnetic stir bar. The emulsion was bubbled with CO_2_ (90 mL·min^−1^) for 20 min with stirring. Later, the unstable colloid solution was sparged with N_2_ (90 mL·min^−1^) for 20 min to remove the CO_2_.

### 2.4. Characteristics

The conductivities of the solutions containing oligochitosan were tested using a conductivity meter (FE30, Mettler Toledo, Columbus, OH, USA) with a quartz conductivity probe at 25 ± 0.5 °C.

The molecular weight of the polymer was measured using gel permeation chromatography (GPC) apparatus (Waters, Milford, MA, USA) equipped with a Waters 1515 pump. During the experiments, pure tetrahydrofuran was selected as the eluent.

The surface tension was determined with the Wilhelmy plate technique via a surface tensiometer (Krüss K100). Several series of constant surface tension data were obtained at 25.0 ± 0.5 °C. Pure water was used to continuously dilute stock aqueous solutions of surfactants, and the surface tension at each concentration was determined by averaging three measurements [17]. To calculate the critical micelle concentration (CMC) values, a plot was drawn to show the linear portions of the surface tension against the logarithm of the concentration, with the CMC data acquired from the x-value of the inflection point in the plot.

A Malvern Mastersizer 2000 (size range of 0.05–2000 nm) was utilized to determine the particle size. A Zetasizer Nano ZS (Malvern Instruments, Malvern, UK) was used to measure the zeta potential of the polymer particles [16].

A Hitachi H600 transmission electron microscope (TEM, Hitachi, Tokyo, Japan) was operated at an accelerating voltage of 75 kV for microscopic observations of the polymer particles [18].

The viscosity of polymer latexes, emulsions and crude oil was tested with an MCR 302 rotational rheometer (Anton Paar, Graz, Austria) equipped with a Searle-type concentric cylinder geometry (CC27).

The value of pH was measured by a Sartorius PB-10 basic pH meter.

## 3. Results and Discussion

### 3.1. CO_2_-Switchable Properties of Oligochitosan

**Scheme 2:**
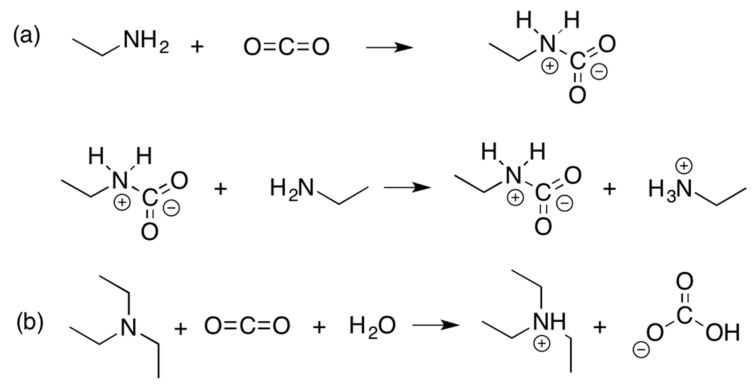
Mechanisms for reaction of CO_2_ with (**a**) primary and (**b**) tertiary amines. Reprinted from [19], with permission from Elsevier 2011.

The molecular structure of oligochitosan is shown in Scheme 1. Oligochitosan has no tertiary amines but has primary amine groups, which can also react reversibly with CO_2_. The combination of CO_2_ and amines can be described by several different mechanisms. The consolidation of CO_2_ and tertiary amines causes the formation of bicarbonate. However, the reaction of primary and secondary amines with CO_2_ is based on a different mechanism. CO_2_ can directly react with primary and secondary amines to produce carbamates through the composition of zwitterionic intermediates [19]. Scheme 2 depicts the CO_2_ reaction mechanisms with primary and tertiary amines. In the presence of CO_2_ in a water solution, the primary amine groups of oligochitosan can be protonated and charged.

**Figure 1:**
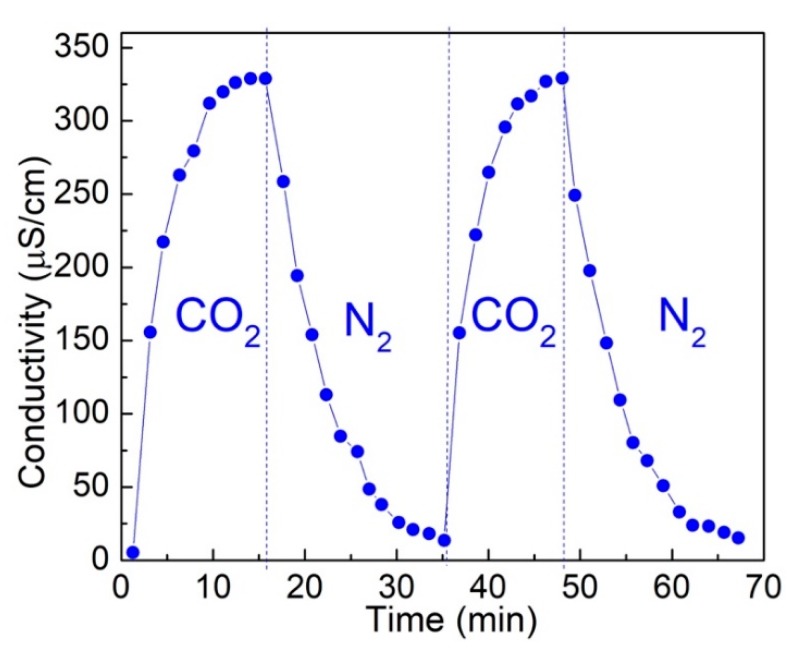
Conductivity of aqueous solution of oligochitosan (0.2 wt.%) against time during two cycles of sparging CO_2_ and N_2_ at 25 ± 0.5 °C.

The conductivity of oligochitosan (0.2 wt.%) in an aqueous solution was tested at 25 ± 0.5 °C for two cycles of switching the sparging of CO_2_ and N_2_. The continuous sparging of CO_2_ caused the solutions to become saturated with CO_2_. The bubbling of N_2_ ensured the removal of CO_2_, and the resultant neutralization of the zwitterionic intermediate salts was accelerated. Figure 1 displays the conductivity measurement results after the alternate bubbling of CO_2_ and N_2_. The conductivity of the aqueous solution of oligochitosan was enhanced from 0.2 to 331 μS/cm through 20 min of the CO_2_ bubbling process. It decreased to its original value after the N_2_ bubbling. The unprotonated oligochitosan turned into a carbamate salt in the presence of water and CO_2_. It is obvious that the aqueous solutions of oligochitosan had considerable switchability because there were primary amine groups in their molecular structures (Figure 1).

### 3.2. Reversible Emulsion

Emulsification is a competent approach for diminishing the viscosity of crude oil. The issues encountered in pipeline transportation could be solved if the crude oil emulsions had CO_2_ switchability. Crude oil emulsions that have lower viscosity with CO_2_ switchable surfactants could be prepared for transport and the crude oil and water could be detached after transporting through the removal or addition of CO_2_. If the surface activity of common surfactants in aqueous solution could be reversibly modified by switchable water and CO_2_, then an emulsion stabilized by such surfactants could have reversible stability controlled by the switchable water and CO_2_.

**Figure 2:**
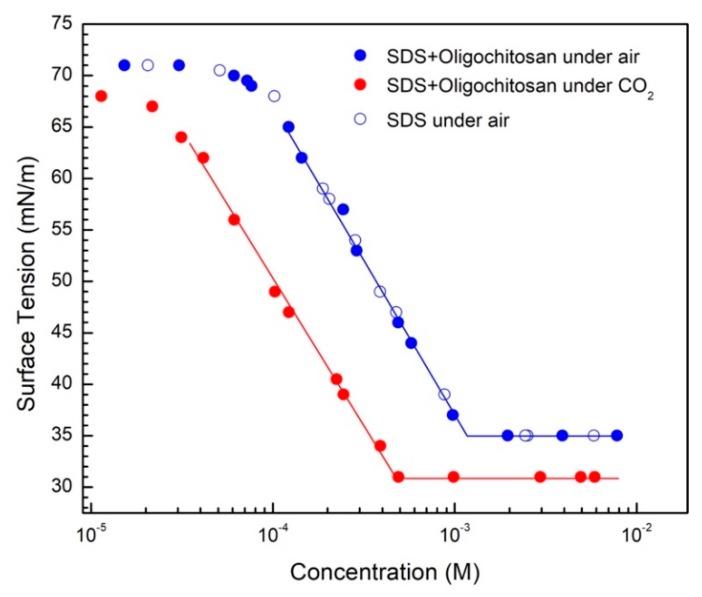
Surface tension against concentration of aqueous solutions of sodium dodecyl sulfate (SDS) and oligochitosan in presence of air or CO_2_ at 25 ± 0.5 °C. The plot of an aqueous solution of pure SDS is also shown.

Figure 2 presents data for the surface tension and critical micelle concentration (CMC). The aqueous solutions contained sodium dodecyl sulfate (SDS) and lecithin in the absence and presence of the switchable water additive, oligochitosan, and/or CO_2_. The observed CMC value for SDS showed a good match with the literature values [20]. It was proven that neutral oligochitosan could not change the surface tension and CMC values. The CMC of the pure SDS aqueous solution in the presence of CO_2_ was marginally lower than the value under air because the ionization of the oligochitosan by the CO_2_ dissolved in the water produced carbamate ions, which decreased the surface tension [21,22]. However, the combination of CO_2_ and oligochitosan ensured the appearance of the organic salt and consequently enhanced the ionic strength, resulting in a decrease in the CMC of SDS (Figure 2). The presence of CO_2_ increased the ionic strength of the solution, which decreased the electrostatic repulsion among the intermolecular head groups of the SDS surfactants [22]. After the CO_2_ was removed with N_2_, the CMC value reverted to its original state, because the charged oligochitosan was converted to its neutral form and could not decrease the electrostatic repulsion among the head groups of the SDS surfactants. Consequently, the aqueous solution with dissolved SDS possessed a switchable surface tension behavior, with the help of the CO_2_ and oligochitosan, which acted as a switchable organic salt.

**Figure 3:**
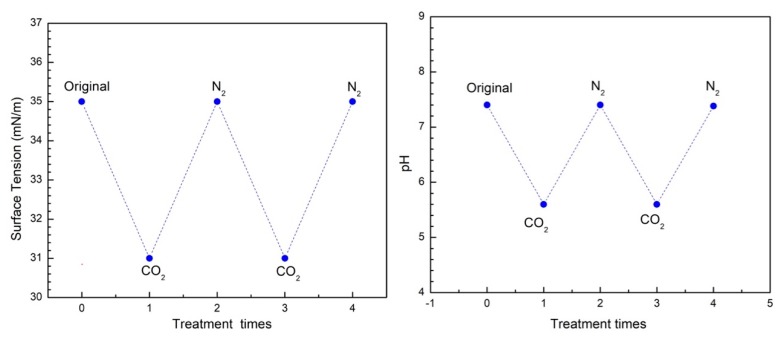
Surface tension (left) and pH value (right) of the aqueous solution of SDS (0.012 mol·L^−1^) with oligochitosan (0.2 wt.%) at 25 ± 0.5 °C against time during two cycles of sparging CO_2_ and N_2_.

Clearly, the surface tension of an SDS aqueous solution can be significantly changed by the CO_2_ trigger after the addition of oligochitosan. Furthermore, the response of the surface tension is reversible. The surface tension of an SDS aqueous solution containing oligochitosan was measured at 25 ± 0.5 °C for two cycles of alternating CO_2_ and N_2_ bubbling (Figure 3). The N_2_ was sparged to facilitate the removal of CO_2_ from the aqueous solution and the subsequent neutralization of the carbamate salts. Figure 3 shows the changeable surface tension and pH value during two cycles of alternating CO_2_ and N_2_ bubbling. The surface tension decreased from 34.9 to 31.0 mN·m^−1^ after the addition of CO_2_ for 20 min. The pH value changed between 7.2 and 5.6 in the CO_2_/N_2_ bubbling cycles. It returned to the original value after bubbling N_2_ through the aqueous solution.

**Figure 4:**
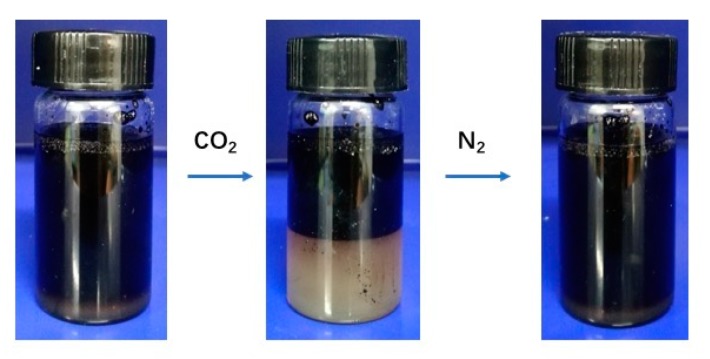
Reversible stability of emulsion of crude oil and aqueous solution. The aqueous solution (5 mL) contains SDS (0.012 mol·L^−1^) and oligochitosan (0.2 wt.%). The original emulsion (left) is stable and oligochitosan does not spoil its stability. The phase separation appears with the addition of CO_2_ (center). However, a stable emulsion is reformed after sparging N_2_ and hand shaking (right).

The results obtained for the switching ability of the oligochitosan show that switching behaviors should also be possible for an emulsion with dissolved oligochitosan. To prove the ability of the oligochitosan and SDS in relation to emulsion stability, a crude oil-in-water emulsion (with a weight ratio of 0.8:1) was prepared using oligochitosan (0.2 wt.% compared to water) and SDS (0.012 mol·L^−1^). Under these circumstances, the SDS with neutral oligochitosan was shown to be an impressive emulsifying agent. The reason for stable emulsion is due to the diffuse electrical double layer surrounding the emulsion droplets, where the inner layer is composed of SDS adsorbed on the surface of droplets and the outer layer is composed of sodium ions, which are attracted to the surface charge. Without CO_2_, the neutral oligochitosan does not adversely influence the electrical double layer. Figure 4 shows that after CO_2_ sparging for 20 min, the crude oil-in-water emulsion was stable for more than 36 h. However, in the presence of CO_2_, oligochitosan reacted by CO_2_ to obtain the charged oligochitosan, increasing the ionic strength, collapsing the electrical double layer, and allowing the droplets to aggregate together. The stability of the emulsion declined sharply through the addition of salt because of the charged oligochitosan due to CO_2_, and the emulsion droplets coalesced over 20 min (Figure 4). Both phases were completely apparent after a further 20 min. However, if N_2_ was sparged into the mixture containing the two phases of the crude oil/water to induce the removal of CO_2_, the primary amine groups of oligochitosan transformed back into their neutral form, and a stable emulsion could be prepared with subsequent stirring.

**Figure 5:**
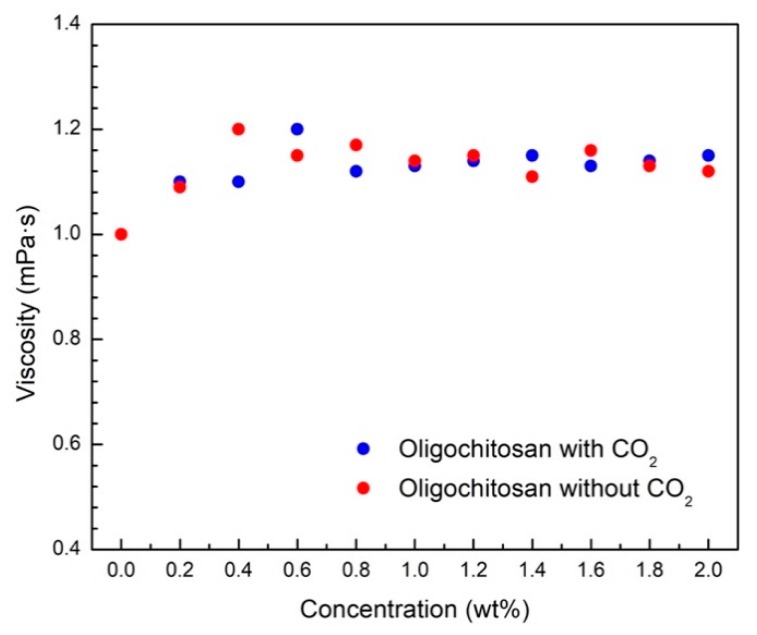
Comparison of apparent viscosity plotted as a function of concentration for oligochitosan solution in the absence or presence of CO_2_ (25 °C, $\dot{\gamma }$ = 10 s^−1^).

In the presence of CO_2_, oligochitosan is a charged polymer, whose rheology would have an impact on colloid systems [23,24,25]. Figure 5 demonstrates the viscosity of oligochitosan aqueous solutions with or without CO_2_. The solutions of oligochitosan (0–2 wt.%) have a viscosity similar to water, namely around 1.2 mPa·s. The reason for this is, as a kind of oligomer, oligochitosan has a low molecular weight. The viscosity of crude oil in this experiment was about 27 mPa·s at 25 ± 0.5 °C, and the emulsion of crude oil had the much lower viscosity than the unemulsified crude oil. The addition of oligochitosan did not influence the viscosity of the emulsion; all the stable emulsions had similar viscosities, ranging from 2.1 to 2.5 mPa·s, including the emulsion that was reformed after N_2_ sparging and hand shaking.

It was reported that the alternate CO_2_/N_2_ bubbling could be utilized to switch an emulsion into two phases, supplying the possibility to trigger the emulsion phase behavior within the oil reservoir by playing with CO_2_ injection [23]. Thus, in the same way, the stabilizing property of the emulsion with oligochitosan was switched off and on through the addition and removal of CO_2_, and it has the potential to be used in the switchable emulsification of crude oil and water. In future, our group will focus on how oligochitosan and CO_2_ affect the combination of non-ionic surfactants and anionic surfactants, as their mixture is used more widely in the real application, such as crude oil recovery.

### 3.3. Switchable Salt Addition of Polystyrene Latex

**Figure 6:**
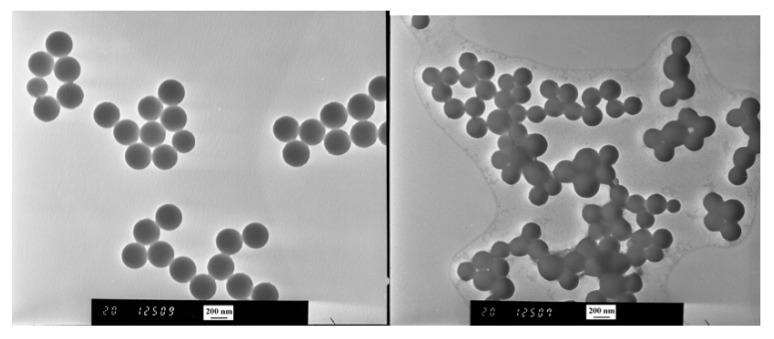
Left transmission electron microscope (TEM) image shows initial PS latex and the right image displays aggregated polystyrene (PS) latex.

A transmission electron microscope (TEM) was used to observe the initial and aggregated PS latexes. The left image in Figure 6 shows a TEM image of particles in the initial PS latex where neutral oligochitosan was dissolved. The TEM image shows that the mean diameter of the particles was approximately 200 nm, and that the oligochitosan had no influence on the PS latex. The right image in Figure 6 confirms that the particles aggregate into clusters, demonstrating that the stability of the latex particles was destroyed by the charged oligochitosan.

**Figure 7:**
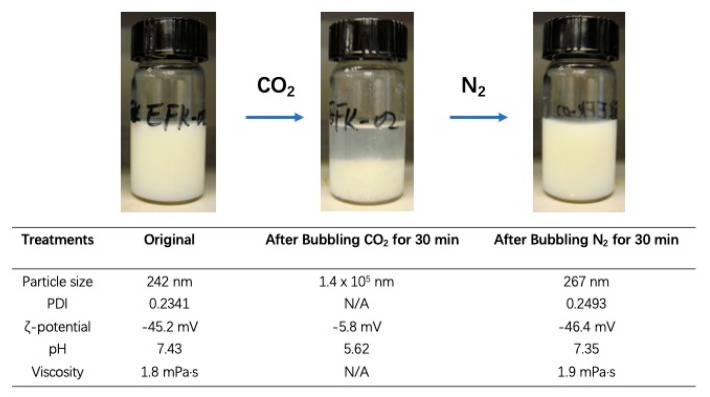
PS latex shows reversible behavior between aggregation and redispersion. Oligochitosan (0.15 g) was added to the PS latex (20 mL, left image), and then CO_2_ (at a flow rate of 90 mL·min^−1^ at 25 ± 0.5 °C for 20 min) was added (central image). Finally, N_2_ (at a flow rate of 90 mL·min^−1^ at 25 ± 0.5 °C for 20 min) was sparged into the latex (right image).

For further confirmation of the TEM findings, the particle size distribution and the surface potential of the nanoparticles were appraised using static light scattering (SLS) and ζ-potential measurements. Figure 7 introduces and analyzes the particle sizes of the PS latexes under different situations at 25 °C. The SLS measurements showed that the diameter of the particles dispersed in the aqueous sample were 242 nm on average, or approximately 10–30 nm larger than the values estimated from the TEM images. This disparity is a result of the disparity between the measurement methods: SLS is used to analyze the particle size of an aqueous solution, and TEM is only used to examine dry particles.

The PS latex had an initial zeta potential of −45.2 mV, suggesting that there were many SDS molecules with negative charges located near the surface of the PS particles. The electrical double layers of the particles were not influenced by the dissolved oligochitosan. However, in the presence of CO_2_, the oligochitosan turned into carbamate salt, which caused the ionic strength to increase and the electrical double layer to collapse. Meanwhile, the value of pH in the latex decreased to 5.62 from 7.43. The zeta potential changed to −5.8 mV from −45.2 mV after bubbling with CO_2_, proving that the PS particles lost their stability and aggregated together. The particle size increased to 140 μm. N_2_ was then sparged through the solution. The zeta potential returned to −46.4 mV, and the white precipitate formed by the aggregated latex was redispersed by hand shaking, which demonstrated the repeatability of the aggregation/redispersion cycle. The particles in the PS latex were successfully aggregated in the presence of CO_2_ and redispersed in the absence of CO_2_. The viscosity of the redispersed latex was 1.9 mPa·s, which was almost the same as the original PS latex.

The above results indicated that the reversible and controlled dispersion/aggregation process was achieved during the CO_2_/N_2_ bubbling. Figure 6 shows images of the latexes at various stages after treatment. The latex aggregated when CO_2_ was added, showing that the colloidal stability was inadequate as a result of the charged oligochitosan. Additionally, the latex could be redispersed after the CO_2_ was removed from the mixture of water and aggregated PS particles, and the amine groups returned to a neutral state.

## 4. Conclusions

An aqueous solution of oligochitosan is stimulus responsive. The conductivity of the aqueous solution of oligochitosan (0.2 wt.%) was switched from 0.2 to 331 μS/cm through the addition and removal of CO_2_. The aqueous solution of oligochitosan has a low viscosity (1.2 mPa·s), which is not influenced by CO_2_. Aqueous solutions of oligochitosan in water had no effect on an emulsion or polymer latex, but exposing the solution to CO_2_ triggered a change in the ionic strength of the aqueous solution, which caused a salt addition behavior. Oligochitosan was utilized to reversibly break a crude oil emulsion into two phases. Moreover, PS latexes could also be reversibly stabilized, which was controlled using CO_2_ when oligochitosan was dissolved in the aqueous phase. In the absence and presence of CO_2_, the zeta potential of the PS latex changed between −5.8 mV and −45.2 mV and diameter of the PS particles dispersed in the aqueous sample fluctuated between 242 nm and 140 μm. The key factor for all of the switching behaviors was controlling the reversible ionic strength using CO_2_. The versatility and sustainability of this concept using natural oligochitosan may successfully prompt a vast amount of future research with potential industrial utility.
